# Risk Factors for Epilepsy After Thrombolysis for Ischemic Stroke: A Cohort Study

**DOI:** 10.3389/fneur.2019.01256

**Published:** 2020-01-23

**Authors:** Rosane Brondani, Andrea Garcia de Almeida, Pedro Abrahim Cherubini, Thaís Leite Secchi, Marina Amaral de Oliveira, Sheila Cristina Ouriques Martins, Marino Muxfeldt Bianchin

**Affiliations:** ^1^Graduate Program in Medicine: Medical Sciences, Universidade Federal Do Rio Grande Do Sul, Porto Alegre, Brazil; ^2^Basic Research and Advanced Investigations in Neurology, Hospital de Clínicas de Porto Alegre, Universidade Federal Do Rio Grande Do Sul, Porto Alegre, Brazil; ^3^Division of Neurology, Hospital de Clínicas de Porto Alegre, Porto Alegre, Brazil; ^4^CETER–Center for Epilepsy Surgery, Hospital de Clínicas de Porto Alegre, Porto Alegre, Brazil

**Keywords:** reperfusion therapy, post-stroke epilepsy, acute seizures, stroke outcome, rt-PA

## Abstract

The effects of thrombolysis in seizure and epilepsy after acute ischemic stroke have been poorly explored. In this study, we examine risk factors and consequences of intravenous rt-PA for treatment of acute ischemic stroke. In a retrospective cohort study we evaluate risk factors for seizure and epilepsy after stroke thrombolysis, as well as the impact of seizures and epilepsy in outcome of stroke patients. In our cohort, mean age of patients was 67.2 years old (*SD* = 13.1) and 79 of them (51.6%) were male and. Initial NIHSS mean score were 10.95 (*SD* = 6.25). Three months NIHSS mean score was 2.09 (*SD* = 3.55). Eighty seven (56.9%) patients were mRS of 0–1 after thrombolysis. Hemorrhagic transformation was observed in 22 (14.4%) patients. Twenty-one (13.7%) patients had seizures and 15 (9.8%) patients developed epilepsy after thrombolysis. Seizures were independently associated with hemorrhagic transformation (OR = 3.26; 95% CI = 1.08–9.78; *p* = 0.035) and with mRS ≥ 2 at 3 months after stroke (OR = 3.51; 95% CI = 1.20–10.32; *p* = 0.022). Hemorrhagic transformation (OR = 3.55; 95% CI = 1.11–11.34; *p* = 0.033) and mRS ≥ 2 at 3 months (OR = 5.82; 95% CI = 1.45–23.42; *p* = 0.013) were variables independently associated with post-stroke epilepsy. In our study, independent risks factors for poor outcome in stroke thrombolysis were age (OR = 1.03; 95% CI = 1.01–1.06; *p* = 0.011), higher NIHSS (OR = 1.08; 95% CI = 1.03–1.14; *p* = 0.001), hemorrhagic transformation (OR = 2.33; 95% CI = 1.11–4.76; *p* = 0.024), seizures (OR = 3.07; 95% CI = 1.22–7.75; *p* = 0.018) and large cortical area (ASPECTS ≤ 7) (OR = 2.04; 95% CI = 1.04–3.84; *p* = 0.036). Concluding, in this retrospective cohort study, the neurological impairment after thrombolysis (but not before) and hemorrhagic transformation remained independent risk factors for seizures or post-stroke epilepsy after thrombolysis. Moreover, we observed that seizures emerged as an independent risk factor for poor outcome after thrombolysis therapy in stroke patients (OR = 3.07; 95% CI = 1.22–7.75; *p* = 0.018).

## Introduction

Stroke is a prevalent disorder, responsible for 9.5% of total deaths each year and is the leading cause of disability in the world (1, 2). After stroke, new onset epilepsy might affect a significant proportion of patients and due to large stroke prevalence, post-stroke epilepsy is one of the leading causes of new diagnoses of epilepsy in patients older than 65 years old (3–6). Seizures or post-stroke epilepsy adds additional burden to stroke patients (7). Both seizures and post-stroke epilepsy might increase stroke morbidity and mortality (8–14). Drugs used for epilepsy control might decrease rehabilitation and impair patient cognition, perhaps having impact in the quality of life of these patients (15–17).

Since the 1995 NINDS study, recombinant tissue plasminogen activator (rt-PA) has been indicated to treat acute ischemic stroke (AIS) (18). Thrombolysis treatment is associated with reduction of disability as measured by the modified Rankin Scale (mRS) (19). The ECASS (20–22), ATLANTIS (23, 24), and EPITHET (25) trials, alone or grouped in a systematic review with meta-analysis (26), confirmed benefits of stroke thrombolysis. Thrombolytic therapy, associated with development of stroke units (27) and specialization in stroke treatment (28, 29) improved stroke outcome. Thrombolysis therapy had impact not only in stroke disability as evaluated by mRS, but it improved overall quality of life in patients. In spite of being largely studied, some effects of reperfusion therapy have not been properly investigated. This is the case of acute seizure after stroke or post-stroke epilepsy, its incidence and its characteristics. In early times, relatively few works have investigated the development of seizure or epilepsy in patients submitted to thrombolysis (30–35). Even fewer studies have evaluated risk factors for acute seizures or post-stroke epilepsy and its characteristics after thrombolysis. While some authors have observed that rt-PA *per se* might have a protective impact on cerebral tissue (36) and acute seizures might be a marker of successful reperfusion (30, 37), others point out that rt-PA might be neurotoxic or might be a risk factor for post-stroke seizures (38). Moreover, Tan et al. designed one study that combined experimental and clinical evidence specifically projected to address the effect of thrombolysis in post-stroke seizures (34). By using transgenic mice and studying clinical thrombolysis, the authors concluded that overexpression of endogenous t-PA lowers seizure threshold, but does not influence epileptogenesis or the development of acquired post-stroke epilepsy. However, considering all these evidences, the impact of thrombolysis therapy in acute seizures or post-stroke epilepsy remains not fully understood and because of its importance, needs to be further studied.

Here we report results of a retrospective cohort-study of 153 patients submitted to thrombolysis therapy for ischemic stroke, having evaluated risk factors for seizures or epilepsy after AIS thrombolysis. We hope this study helps clarify the incidence rates and characteristics of acute seizures or post-stroke epilepsy in patients receiving thrombolysis therapy for treatment of stroke, also aiding in planning new strategies for reperfusion therapies for acute ischemic stroke.

## Methods

### Ethic Statement

Protocol of study was approved by the Ethics Committee of Hospital de Clínicas de Porto Alegre and it was conducted according to the principles expressed in the Declaration of Helsinki. All patients or legal representatives gave written informed consent to participate in this study.

### Study Population

After approval by the Ethics Committee of Hospital de Clinicas de Porto Alegre, we retrospectively evaluated 153 consecutive patients submitted to stroke thrombolysis from 2005 to 2011. After stroke patients were followed for at least 2 years. Among variables studied were age, sex, ethnicity, hypertension, diabetes mellitus, hypercholesterolemia, smoking, drinking alcohol, atrial fibrillation, and variables associated with stroke like National Institute of Health and Stroke Scale (NIHSS), stroke etiology, stroke vascular territories, stroke severity, hemorrhagic transformation, and thrombolysis outcome.

Patients were classified in three groups, according to the presence or absence of early-onset seizures (i.e., if seizures developed during the first 7 days after stroke), late onset-seizures (i.e., if seizures started after the first week), and post-stroke epilepsy. Post-stroke epilepsy was independent of the time of seizures onset, encompassing both types of patients; those patients with earlier seizures that continued to have seizures after the acute phase, as well as those patients that presented seizures after the first week after stroke. Thus, post-stroke epilepsy was considered if patients had recurrent seizures, showed seizures after attempts to withdraw antiepileptic drugs, or if patients were maintained in treatment with anti-epileptic drugs because of a great chance of seizure recurrence as evaluated by EEGs, neuroimaging findings, and physician evaluation. As a consequence, post-stroke epilepsy was defined according to the recommendations of the International League Against Epilepsy (39). Moreover, all patients were submitted to at least two EEG monitoring for at least 30 min. All patients had seizures of focal onset and thus seizures were classified as focal seizures. As video-EEG was not performed, further characteristics of the seizures (e.g., semiology) could not be precisely established. Patients with no seizures were compared to those who presented early seizure, late seizures or post stroke epilepsy in order to determine risk factors for development of seizures or post-stroke epilepsy and for studying other plausible associations.

### Thrombolysis Protocol and Patients Evaluation

Thrombolytic treatment was administered following the American guideline to thrombolysis in stroke (19). Briefly, patients received an rt-PA dose of 0.9 mg/kg within to 3 or 4.5 h from the onset of symptoms. Thrombolytic drug was administered over a period of 1 h to patients older than 18 years of age with a clinical diagnosis of AIS, screened by computed tomography (19). Stroke severity was evaluated using the NIHSS, a scale of 0–42 points, where minimal or no symptoms are scored as 0 and points are added according to increasing stroke severity. NIHSS scale was validated for the Brazilian population and applied by a neurologist (40). NIHSS results were recorded before treatment and 3 months later. After thrombolysis, patients were admitted at the stroke unit or the intensive care unit. Response to treatment was evaluated according to the Rankin scale (41) and NIHSS scale 3 months after stroke. For analysis, a good outcome was considered if patients showed mRS scores of 0 or 1 and a bad outcome was defined as mRS of 2 or above. Bleeding was defined as any central nervous system bleeding, defined according to radiological criteria (42).

### Statistical Analysis

Categorical data were compared using Fisher's exact test, and results are expressed in odds ratio with 95% confidence interval. Numerical variables were compared by Student's independent *t*-test. This allowed identification of potential prognostic indicators. Variables with a significance level of *p* < 0.20 or lower on initial univariate analysis were included in a multivariate Cox proportional hazards regression model. This method allows testing the correlation of specific variables with outcome while taking into account any interactions and associations among those variables, and their variation with time. All results were considered significant if *p* < 0.05. The software SPSS version 20.0 was used for statistical analysis.

## Results

The characteristics of 153 patients included in the study are presented in Table 1. Seventy-four patients 74 (48.4%) were female and mean age of patients was 67.2 years-old (*SD* = 13.1). Frequency of vascular risk factors for stroke, among them hypertension, diabetes mellitus, hypercholesterolemia, smoking, drinking alcohol, and atrial fibrillation are also present in Table 1. NIHSS mean score was, 10.95 (*SD* = 6.25) and 2.09 (*SD* = 3.55) after 3 months. Hemorrhagic transformation occurred in 22 (14.4%) patients. A good outcome, defined by mRS of 0–1, was observed in 87 (56.9%) patients.

**Table 1:** Characteristics of 153 patients treated with rt-PA.

**Variables**	**N°**	**(%)**
Age in years, mean (SD)	67.24	(13.13)
Female sex	74	(48.4%)
White ethnicity	140	(91.5%)
**Vascular risk factors**		
Hypertension	129	(84.3%)
Diabetes mellitus	36	(23.5%)
Hypercholesterolemia	65	(42.5%)
Smoking	52	(34.0%)
Drinking alcohol	16	(10.5%)
Atrial fibrillation	50	(32.7%)
Previous stroke	42	(27.5%)
Baseline NIHSS, mean (SD)	10.95	(6.25)
Systolic blood pressure, mmHg (SD)	159.59	(34.6)
Diastolic blood pressure, mmHg (SD)	91.42	(19.5)
Glucose level (mg/dl) (SD)	139.4	(61.9)
**Stroke etiology**		
Cardioembolism	66	(43.1%)
Large-artery atherosclerosis	52	(34.0%)
Small-artery occlusion	3	(2.0%)
Other	23	(15.0%)
Undetermined	9	(5.9%)
**Occlusion location**		
Anterior circulation	121	(79.1%)
Posterior circulation	31	(20.3%)
Cortical involvement	117	(76.5%)
**Aspects score**		
>7	117	(76.5%)
≤7	36	(23.5%)
Hemorrhagic Transformation (HT)	22	(14.4%)
Outcome		
NIHSS 3 months, mean (SD)	2.09	(3.55)
Rankin score 3 months		
0–1	87	(56.9%)
2–6	66	(43.1%)
Mortality	44	(28.8%)

Of 153 patients, 21 (13.7%) presented seizures. Seizures were classified as focal with aware and motor onset with clonic facial and arm movements in two patients. Eleven patients had focal seizures, with impaired awareness and motor onset with clonic facial and limb movements with posterior evolution to bilateral tonic-clonic seizures. Additionally, five patients had focal seizures with impaired awareness with no motor onset that evolved to bilateral tonic-clonic seizures. In three patients, the seizure onset was unknown and patients evolved possibly to bilateral tonic-clonic seizures, if one is considering that the onset was probably focal. Table 2 presents distribution of seizures according to variables studied. Seizures were more prone to occur in patients with diabetes, in those with higher NIH stroke scores at first evaluation, and also after 3 months. AIS that involved cortical areas or those with hemorrhagic transformation were also more frequently associated with seizures. No significant differences were observed in the other parameters investigated. Moreover, seizures could not be attributed to renal dysfunction or blood sodium abnormalities. In patients that had seizures, the mean blood creatine 24 h before seizures was 1.03 mg/dl (minimum = 0.4 mg/dl and maximum = 1.9 mg/dl; *SD* = 0.32), the mean blood urea was 40.76 mg/dl (minimum = 24 mg/dl and maximum = 60 mg/dl; *SD* = 11.09), and the mean blood sodium was 140.38 mEq/L (minimum = 135.00 mEq/L and maximum = 145.00 mEq/L; *SD* = 2.49). These values were not different from those of patients that had no seizures. As stroke thrombolysis outcome 3 months later was measured using NIHSS and also mRS, only mRS, dichotomized in favorable or unfavorable outcome, was included in the regression model.

**Table 2:** Variables according with seizures.

**Variables**	**Seizures (%) (*n* = 21)**	**No seizures (%) (*n* = 132)**	**OR**	**95% CI**	***p***
Age in years, mean (SD)	67.05 (10.8)	67.27 (13.5)	–	–	0.94
Female sex	10 (47.6%)	64 (48.5%)	0.97	0.38–2.43	1.00
White ethnicity	19 (90.5%)	121 (91.7%)	1.16	0.24–5.63	0.69
Vascular risk factors					
Hypertension	19 (90.5%)	110 (83.3%)	1.90	0.41–8.75	0.53
Diabetes mellitus	10 (47.6%)	26 (19.7%)	**3.71**	**1.42–9.66**	0.01^*^
Hypercholesterolemia	12 (57.1%)	53 (40.2%)	1.99	0.78–5.05	0.16
Smoking	7 (33.3%)	45 (34.1%)	0.97	0.36–2.57	1.00
Drinking alcohol	1 (4.8%)	15 (11.4%)	0.39	0.05–3.12	0.70
Atrial fibrillation	9 (42.9%)	41 (31.1%)	1.66	0.65–4.26	0.32
Previous Stroke	4 (19.0%)	38 (28.8%)	0.58	0.18–1.84	0.44
Baseline NIHSS, mean (SD)	13.81 (6.79)	10.49 (6.06)	–	–	0.02^*^
Systolic blood pressure, mmHg (SD)	172.71 (38.51)	157.51 (33.63)	–	–	0.06
Diastolic blood pressure, mmHg (SD)	94.24 (21.06)	90.98 (19.23)	–	–	0.48
Glucose level (mg/dl) (SD)	164.1 (85.09)	135.45 (56.77)	–	–	0.05
Stroke etiology			–	–	0.44
Cardioembolism	9 (42.9%)	57 (43.2%)			
Large-artery atherosclerosis	10 (47.6%)	42 (31.8%)			
Small-artery occlusion	0	3 (2.3%)			
Other	2 (9.5%)	21 (15.9%)			
Undetermined	0	9 (6.8%)			
Cortical involvement	21 (100.0%)	96 (72.7%)	–	–	0.004^*^
Aspects score			0.50	0.14–1.81	0.41
>7	18 (85.7%)	99 (75%)			
≤7	3 (14.3%)	33 (25%)			
Hemorrhagic transformation	7 (33.3%)	15 (11.4%)	**3.90**	**1.36–11.20**	0.02^*^
Outcome					
NIHSS 3 months, mean (SD)	5.38 (5.20)	1.46 (2.74)	–	–	<0.001^*^
Rankin score−3 months			**3.97**	**1.45–10.90**	0.008^*^
0–1	6 (28.6%)	81 (61.4%)			
2–6	15 (71.4%)	51 (38.6%)			
Mortality	3 (14.3%)	41 (31.1%)	0.37	0.10–1.33	0.13

*Statistically significant differences are highlighted in bold*.

* *Indicate the values are statistically significant*.

Table 3 presents distribution of patients according with presence of early or late seizures. Four patients showed seizures during first 7 days after stroke (early seizures) while 17 patients showed seizures after 7 days of the stroke (late seizures). Interestingly, those patients who presented early seizures showed a trend to better thrombolysis outcome. In spite of this, no significant differences were observed in the parameters investigated (Table 3).

**Table 3:** Variables according with early or late seizures.

**Variables**	**Early seizures (%) (*n* = 4)**	**Late seizures (%) (*n* = 17)**	**OR**	**95% CI**	***p***
Age in years, mean (SD)	73 (11.94)	65.65 (10.40)	–	–	0.23
Female sex	2 (50.0%)	8 (47.1%)	0.89	0.10–7.86	1.00
White ethnicity	3 (75.0%)	16 (94.1%)	0.19	0.01–3.90	0.35
Vascular risk factors					
Hypertension	4 (100.0%)	15 (88.2%)	1.27	1.00–1.60	1.00
Diabetes mellitus	1 (25.0%)	9 (52.9%)	3.38	0.29–39.32	0.59
Hypercholesterolemia	1 (25.0%)	11 (64.7%)	5.50	0.46–65.16	0.27
Smoking	1 (25.0%)	6 (35.3%)	1.64	0.14–19.39	1.00
Drinking alcohol	0	1 (5.9%)	0.80	0.64–1.00	1.00
Atrial fibrillation	0	9 (52.9%)	0.67	0.45–1.00	0.10
Previous stroke	2 (50.0%)	2 (11.8%)	0.13	0.01–1.55	0.15
Baseline NIHSS, mean (SD)	8.3 (4.57)	15.1 (6.65)	–	–	0.07
Systolic blood pressure, mmHg (SD)	202.0 (42.61)	165.8 (35.34)	–	–	0.09
Diastolic blood pressure, mmHg (SD)	86.2 (34.97)	96.1 (17.47)	–	–	0.41
Glucose level (mg/dl) (SD)	150.0 (118.77)	167.4 (79.65)	–	–	0.72
Stroke etiology					
Cardioembolism	0	9 (52.9%)			
Large-artery atherosclerosis	3 (75.0%)	7 (41.2%)			
Other	1 (25.0%)	1 (5.9%)			
Aspects score			0.78	0.61–1.00	1.00
>7	0	3 (17.6%)			
≤7	4 (100.0%)	14 (82.4%)			
Hemorrhagic Transformation	0	7 (41.2%)	0.71	0.51–1.00	0.26
Outcome					
NIHSS 3 months, mean (SD)	2.3 (3.86)	6.1 (5.29)			0.19
Rankin score 3 months			14.0	1.06–185.49	0.053
0–1	3 (75.0%)	3 (17.6%)			
2–6	1 (25.0%)	14 (82.4%)			
Mortality	0	3 (17.6%)	0.78	0.61–1.00	1.00

Of 153 patients, 15 presented epilepsy. Table 4 presents post-stroke epilepsy according to clinical and acute stroke variables studied. Factors associated with post-stroke epilepsy after thrombolysis in AIS were hemorrhagic transformation, involvement of cortical areas, higher NIHSS at stroke onset and after 3 months, and mRS of 2 or higher 3 months after stroke. No significant differences were observed in the other parameters investigated. After Cox regression (Table 5), stroke severity, as defined by mRS 2 or higher 3 months after stroke and hemorrhagic transformation were the only two variables independently associated with seizures. As expected, only patients with cortical involvement presented seizures after stroke. As no one patient with noncortical involvement presented seizures, an odds ratio could not be adequately estimated. Also, including cortical involvement in logistic regression models resulted in multicollinearity and an absurdly high 95% confidence interval. For this reason, we did not include cortical involvement in the Cox regressions analysis. After Cox regression, mRS of 2 or higher 3 months after stroke and hemorrhagic transformation were the only two independent predictors of post-stroke epilepsy (Table 6). The effect of stroke cortical involvement was not included in the model, for reasons explained above, but it is presented as a variable associated with post-stroke epilepsy.

**Table 4:** Variables according epilepsy.

**Variables**	**Epilepsy (%) (*n* = 15)**	**No epilepsy (%) (*n* = 138)**	**OR**	**95% CI**	***p***
Age in years, mean (SD)	64.07 (9.75)	67.58 (13.43)	–	–	0.327
Female sex	6 (40%)	68 (49.3%)	0.69	0.23–2.03	0.591
White ethnicity	13 (86.7%)	127 (92.0%)	1.78	0.35–0.84	0.618
Vascular risk factors					
Hypertension	14 (93.3%)	115 (83.3%)	2.80	0.35–22.36	0.468
Diabetes mellitus	6 (40%)	30 (21.7%)	2.40	0.79–7.28	0.12
Hypercholesterolemia	7 (46.7%)	58 (42.0%)	1.21	0.41–3.52	0.79
Smoking	5 (33.3%)	47 (34.1%)	0.97	0.31–2.99	0.60
Drinking alcohol	1 (6.7%)	15 (10.9%)	0.59	0.07–4.77	1.00
Atrial fibrillation	7 (46.7%)	43 (31.2%)	1.93	0.66–5.67	0.253
Previous stroke	1 (6.7%)	41 (29.7%)	0.17	0.02–1.33	0.070
Baseline NIHSS score, mean (SD)	15 (7.25)	10.51 (5.99)	–	–	0.008^*^
Systolic blood pressure, mmHg (SD)	167.73 (35.24)	158.71 (34.54)	–	–	0.339
Diastolic blood pressure, mmHg(SD)	93.07 (22.85)	91.25 (19.14)	–	–	0.732
Glucose level (mg/dl) (SD)	163.87 (86.98)	136.72 (58.32)	–	–	0.107
Stroke etiology			–	–	0.572
Cardioembolism	7 (46.7%)	59 (42.8%)			
Large-artery atherosclerosis	7 (46.7%)	45 (32.6%)			
Small-artery occlusion	0	3 (2.2%)			
Other	1 (6.7%)	22 (15.9%)			
Undetermined	0	9 (6.5%)			
Cortical involvement	15 (100.0%)	102 (73.9%)	–	–	0.023^*^
Aspects score			0.79	0.21–2.99	1.00
>7	12 (80.0%)	105 (76.1%)			
≤7	3 (20.0%)	33 (23.9%)			
Hemorrhagic transformation	6 (40.0%)	16 (11.6%)	5.08	1.60–16.17	0.009^*^
Outcome					
NIHSS 3 months (SD)	6.47 (5.55)	1.52 (2.76)	–	–	<0.001^*^
Rankin score 3 months			**6.22**	**1.68–23.07**	0.004^*^
0–1	3 (20.0%)	84 (60.9%)			
2–6	12 (80.0%)	54 (39.1%)			

*Statistically significant differences are highlighted in bold*.

* *Indicate the values are statistically significant*.

**Table 5:** Independent risk factors for seizures.

**Variables**	**Crude OR 95%/I**	***p***	**Adjusted OR 95%CI**	***p***
NIHSS on admission	1.08 (1.01–1.15)	0.03	1.02 (0.94–1.11)	0.584
Hemorrhagic transformation	3.90 (1.36–11.20)	0.015	**3.26 (1.08–9.78)**	0.035^*^
mRS ≥ 2	3.97 (1.45–10.90)	0.008	**3.51(1.20–10.32)**	0.022^*^
Glucose on admission	1.07 (1.00–1.01)	0.058	0.99 (0.99–1.00)	0.564
Hypercholesterolemia	1.99 (0.78–5.05)	0.160	0.84 (0.53–1.31)	0.438
Systolic blood pressure at onset	1.01 (1.00–1.03)	0.065	1.01 (0.99–1.02)	0.127
Diabetes mellitus	3.71 (1.42–9.66)	0.010	2.69 (0.86–8.37)	0.088

*Statistically significant differences are highlighted in bold*.

* *Indicate the values are statistically significant*.

**Table 6:** Independent risk factors for epilepsy.

**Variables**	**Crude OR 95%CI**	***p***	**Adjusted OR 95%CI**	***p***
NIHSS on admission	1.10 (1.02–1.19)	0.014	1.05 (0.96–1.49)	0.287
Hemorrhagic transformation	5.08 (1.60–16.17)	0.009	3.55 (1.11–11.34)	0.033^*^
mRS ≥ 2	6.22 (1.68–23.07)	0.004	5.82 (1.45–23.42)	0.013^*^
Glucose on admission	1.01 (1.00–1.01)	0.118	1.00 (0.99–1.00)	0.581
Systolic blood pressure at onset	1.01 (0.99–1.02)	0.338	1.01 (1.00–1.02)	0.108
Diabetes mellitus	2.40 (0.79–7.28)	0.12	0.37 (0.12–1.15)	0.085

*Statistically significant differences are highlighted in bold*.

* *Indicate the values are statistically significant*.

At this point, it was not established if seizures would be an independent risk factor for unfavorable outcome in stroke thrombolysis or, if alternatively, those patients who had larger strokes or those who had poor reperfusion after thrombolysis would be at risk for seizures. To solve this problem, we analyzed if seizures were an independent predictor for unfavorable thrombolysis outcome. After corrections for multiple comparisons, we concluded that seizure is effectively an isolated risk factor for unfavorable outcome after thrombolysis. In this analysis, NIHSS, hemorrhagic transformation, and size of stroke as evaluated by ASPECTS also emerged as independent risk factors for unfavorable outcome in stroke thrombolysis (Table 7).

**Table 7:** Independent risk factor for unfavorable outcome in thrombolysis: the independent effect of seizures.

**Variable**	**mRS 0–1 (*n* = 67)**	**mRS 2–6 (*n* = 66)**	**Crude OR 95% CI**	**Adjusted OR 95%CI**	**Adjusted *p***
Age, mean (SD)	65.21 (14.52)	69.91 (10.56)	1.03 (1.00–1.06)	1.03 (1.01–1.06)	0.011^*^
Systolic arterial pressure, mean (SD)	155.40 (32.82)	165.12 (36.33)	1.01(1.00–1.02)	1.00 (0.96–1.01)	0.484
NIHSS, mean (SD)	8.26 (4.29)	14.48 (6.68)	1.23 (1.15–1.34)	1.08 (1.03–1.14)	0.001^*^
Serum glucose, mean (SD)	127.08 (47.03)	155.61 (74.53)	1.01 (1.00–1.01)	1.00 (0.99–1.00)	0.464
Hemorrhagic transformation	05 (5.7%)	17 (25.8%)	5.69 (1.98–16.39)	2.33 (1.11–4.76)	0.024^*^
Seizure	06 (6.9%)	15 (22.7%)	3.97 (1.45–10.90)	3.07 (1.22–7.75)	0.018^*^
Cortical involvement	63 (72.4%)	54 (81.8%)	1.71 (0.78–3.75)	1.51 (0.73–3.14)	0.266
Large cortical area (ASPECTS ≤ 7)	09 (11.3%)	27 (40.9%)	6.00 (2.57–13.99)	2.04 (1.04–3.84)	0.036^*^
Hypertension	69 (79.3%)	60 (90.9%)	2.61 (0.97–7.00)	0.58 (0.24–1.43)	0.239
Diabetes mellitus	15 (17.2%)	21 (31.8%)	2.24 (1.05–4.79)	0.74 (0.34–1.60)	0.444

*Statistically significant differences are highlighted in bold*.

* *Indicate the values are statistically significant*.

## Discussion

In our cohort, 21 (13.7%) patients had seizures and 15 (9.8%) patients developed epilepsy after thrombolysis for AIS treatment. Seizures were independently associated with hemorrhagic transformation and with mRS ≥ 2 at 3 months after stroke. Early seizures showed a trend to occur more often in patients who showed favorable outcome. Hemorrhagic transformation and unfavorable outcome, as measured by mRS ≥ 2 at 3 months, were variables independently associated with post-stroke epilepsy. An additional analysis confirmed that seizures were an independent factor for poor outcome in stroke thrombolysis.

Post-stroke seizure is a well-studied complication of stroke, but many questions remain to be solved, the main question being what defines early seizures, late seizures, or post-stroke epilepsy. These have been defined in heterogeneous ways in the literature, making it difficult to compare results and variables among studies. Here, we attempt to separate early or late seizures and also to evaluate post-stroke epilepsy. In studies from the last two decades, the frequency of seizures in non-thrombolyzed stroke patients range from 2 to 20% (7, 9, 43–70). In pre-thrombolysis years and after, reported risk factors for post-stroke seizures or epilepsy were multilobar involvement (43), embolic etiology (44), cortical involvement (7, 9, 43, 45, 46), headache and loss of consciousness during stroke (47), either female or male sex (45, 47, 48), hemorrhagic transformation (49), and severity of stroke (7, 11, 50–53). In spite of different variables, some of the parameters studied may reflect similar aspects of the same variable, for example severity of the event, extension of cortical involvement, and hemorrhagic transformation. The lack of modern statistical tools in earlier analysis make difficult for the identification of isolated risk factors for post-stroke seizures or post-stroke epilepsy. In a recent systematic review and meta-analysis, Zhang et al. described as risk factors for early post-stroke seizures hemorrhagic transformation, stroke severity, and alcoholism (54). In this same meta-analysis, risk factors for late seizures were cortical involvement and stroke severity (54). In our study, all patients with seizures or epilepsy had cortical involvement and the inclusion of cortical involvement in logistic regression models resulted in multicollinearity. Cortical involvement in stroke is perhaps better seen as a *sine qua non* condition for seizure or epilepsy to occur rather than as a risk factor such as others. In fact, it is reasonable to expect that virtually all patients with seizure or epilepsy would have some degree of cortical involvement. Although subcortical epilepsy does exist, it is extremely rare and should not affect post-stroke seizures or epilepsy rates. Thus, it is necessary, in the future, to evaluate specific sub-types of cortical regions and the extension of its involvement as risk factors for post-stroke seizures rather than the cortical involvement *per se*.

As large-scale reperfusion therapy is relatively recent, much less is known about seizures or epilepsy after AIS thrombolysis. First reports observed that seizures during or right after thrombolysis could be a marker of successful reperfusion (30, 37). If this would be common, it would be expected that reperfusion therapy would lead to seizure or post-stroke epilepsy increase. After these first observations, some recent studies reported on frequency of seizures or post-stroke epilepsy in AIS thrombolysis era (10, 31–35, 38, 71–78). The frequency of seizures after thrombolysis ranged from 4–15%, and it is in the range of pre-thrombolytic decades. The frequency of early seizures, defined as seizures which occurred during the first 7 days after stroke and thrombolysis, ranged from 2.5 to 5%. The frequency of late seizures, again described as seizures occurring after the first 7 days from stroke, ranged from 1.5 to 11.3% of patients. These studies also evaluated risk factors for seizure development in the thrombolysis scenario. In a case-control study of 28 patients submitted to thrombolysis and 100 controls, Alvarez et al. identified thrombolysis itself and cortical involvement as risk factors for seizures after reperfusion therapy (38). Other risk factors for seizures after stroke thrombolysis were atrial fibrillation (32), younger age, and higher NIHSS at arrival (33). Keller et al. (35) identified as independent risk factors for post-stroke epilepsy low Barthel Index at discharge, hemianopia, infection acquired during the hospital stay, and involvement of the temporal lobe or perirolandic cortex. In this study rt-PA was not an independent risk factor for post-stroke epilepsy. Tan et al. evaluated the role of rt-PA in development of post-stroke epilepsy in a controlled way (34). They study seizures after stroke in mice deficient in t-PA and in animals that overexpress t-PA. These authors also evaluated thrombolysis in a clinical scenario comparing post-stroke epilepsy in patients with or without thrombolysis (34). They concluded that therapeutic administration of t-PA does not influence the development of acquired post-stroke epilepsy. However, other authors have reported an increase in seizures incidence during the acute period of stroke after rt-PA (31, 38). Moreover, Naylor et al. reported that the risk of seizures after rt-PA extends further than the acute stage of stroke, and might occur over a 24-months period (71). Among the mechanisms involved in the effect of rt-PA in post-stroke increasing seizure risk are sudden change in cerebral perfusion which might be associated with a cascade of inflammatory responses, the development of the reperfusion syndrome and subsequent seizures and post-stroke epilepsy. In spite that rt-PA might show some toxicity, the similar rates of occurrence of post-stroke seizures between patients treated only with IA with those treated with IA plus rt-PA suggest that reperfusion, *per se*, might be the responsible for the increase of seizure frequency after rt-PA therapy (71).

In spite of earlier evidences that seizures might be a sign of successful reperfusion (30, 37), some authors suggest that patients who showed seizures after thrombolysis might present worst thrombolysis outcome when compared to patients without seizures (10, 32). In this venue, our results are in line with ENCHANTED trial that showed that seizures after stroke are associated with poor outcome (73). Since studies in post-stroke epilepsy after thrombolysis are few, recent, retrospective in design, and evaluate a relatively small number of patients, their findings need further confirmation. Unfortunately, large randomized controlled multicenter trials for stroke thrombolysis which confirmed the intravenous safety and efficacy of rt-PA for stroke did not report on seizure occurrence or post-stroke epilepsy. The National Institute of Neurological Disorders and Stroke rt-PA Stroke Study Group (18), ECASS (20–22), ATLANTIS (23, 24) EPITHET trials (25), SITS-MOST (79), and SITS-ISTR (80).

Thus, the magnitude of seizure occurrence or post-stroke epilepsy and its impact on patient quality of life remains not fully studied, and prospective studies designed to assess the burden of post-stroke epilepsy in thrombolysis are still necessary. Eventually, these studies would be important for planning and developing new strategies for preventing seizures or epilepsy in AIS reperfusion scenario as well.

Taking all the data together, the clinical risk factors for seizures or post-stroke epilepsy seems to be related to the degree of cortical involvement, the size of the brain region involved and the efficacy of reperfusion. The *sine qua non* condition for post-stroke seizures seems to be related to the cortical involvement itself, for obvious reasons. In our study, neuroimaging of all patients with seizure or epilepsy showed cortical involvement. Secondly, seizures or epilepsy risk might be related to the extension of the cortical involvement. In the DECIMAL trial (DEcompressive Craniectomy In MALignant MCA Stroke), authors observed greater risk of seizures in patients with malignant middle cerebral artery stroke, independently of decompressive craniectomy, being 6 of 15 survivors (40%) with craniectomy and 2 of 4 survivors (50%) without decompressive hemicraniectomy (81). We have previously observed similar rates after decompressive craniectomy (82). Our data showed that initial NIHSS scores correlate with seizures or epilepsy development. However, Cox regression analysis showed that only residual deficits that remained after thrombolysis, as evaluated by mRS unfavorable outcome, were independently associated with seizures or post-stroke epilepsy. This suggests that when reperfusion therapy saves neural tissue, the chance of post-stroke epilepsy decreases. On the other hand, thrombolytic therapy is associated with an increased chance of hemorrhagic transformation in ischemic stroke. Bleeding, on its turn, is associated with increased chance of seizures or post-stroke epilepsy as observed in our study and in line with literature. This is further supported by higher rates of seizures or post-stroke epilepsy after hemorrhagic stroke when compared with ischemic stroke. Thus, patients who experience hemorrhagic transformation after thrombolysis might have an increased risk for seizures or post-stroke epilepsy. Considering hemorrhagic transformation after rt-PA, one might expect increased rates of seizures or post-stroke epilepsy. However, this might not be the case because of the neural tissue saving effects of thrombolysis that might well counterbalance its hemorrhagic effects. These possibilities are interesting matters for future research. Nevertheless, we observed an increased unfavorable outcome in patients who develop clinical seizures. This is an interesting finding, in line with the literature, and should also motivate future research.

We recognize that our work has limitations. This is a retrospective study with a relative small sample size, which resulted in lower precision and confidence interval very long. Moreover, negative associations need to be interpreted with caution because of lack of statistical power. Also, due to the small number of patients included in this study, we could not evaluate if seizures, as well as post-stroke epilepsy were both independent risk factors for poor outcome in ischemic stroke. However, we could observe interesting aspects of seizures or post-epilepsy in the thrombolysis scenario. Some of our findings corroborate the literature, and others are new and need confirmation.

In summary, in our study seizures or post-stroke epilepsy rates after thrombolysis are high, perhaps higher than in patients not submitted to thrombolysis. In our study, hemorrhagic transformation and degree of neurological compromise after thrombolysis, but not before, were the only two variables independently associated with seizures or post-stroke epilepsy. Importantly, we also observed that seizures were an independent risk factor associated with a less favorable outcome after thrombolysis therapy.

## Ethics Statement

Protocol of study was approved by the Ethics Committee of Hospital de Clínicas de Porto Alegre and it was conducted according to the principles expressed in the Declaration of Helsinki. All patients or legal representatives gave written informed consent to participate in this study.

## Author Contributions

RB, AA, PC, TS, MO, SM, and MB: conception and design of the work, acquisition, analysis and interpretation of data for the work, and final approval of the version to be published. RB, TS, and MB: drafting the work and revising the manuscript.

### Conflict of Interest

The authors declare that the research was conducted in the absence of any commercial or financial relationships that could be construed as a potential conflict of interest.
